# Fluorescence-guided bone resection by using Visually Enhanced Lesion Scope in diffuse chronic sclerosingosteomyelitis of the mandible: Clinical and pathological evaluation

**DOI:** 10.4317/jced.52268

**Published:** 2015-10-01

**Authors:** Daigo Yoshiga, Masaaki Sasaguri, Kou Matsuo, Sei Yoshida, Masataka Uehara, Manabu Habu, Kazuya Haraguchi, Tatsurou Tanaka, Yasuhiro Morimoto, Izumi Yoshioka, Kazuhiro Tominaga

**Affiliations:** 1 Department of Science of Physical Function, Division of Maxillofacial Surgery, Kyushu Dental University, Fukuoka, Japan; 2 Department of Health Promotion, Division of Oral Pathology, Kyushu Dental University, Fukuoka, Japan; 3 Department of Oto-Rhino-Laryngology, Graduate School of Medical Sciences, Kyushu University, Fukuoka, Japan; 4 Department of Science of Physical Function, Division of Oral and Maxillofacial Radiology, Kyushu Dental University, Fukuoka, Japan; 5 Department of Science of Physical Function, Division of Oral Medicine, Kyushu Dental University, Fukuoka, Japan

## Abstract

Diffuse chronic sclerosingosteomyelitis (DCSO) is a refractory disease, becausethe etiology and pathogenesis remain poorly understood and to determine the border betweenunhealthy boneandhealthybone is difficult. However, progressive inflammation, clinical symptoms and a high recurrence rate of DCSO were the reasons for surgical treatment. We report a case of a 66-year old woman with DCSO of the right side of mandible who was treated with hemimandibulectomy and simultaneous reconstruction by vascularized free fibula flap. After preoperative administration of minocycline for 1 month, the bone fluorescence was successfully monitored by using a Visually Enhanced Lesion Scope (VELscope®). Intraoperatively, we could determine the resection boundaries. We investigated the clinical and histopathological findings. The fluorescence findings were well correlated with histopathological findings. Using a VELscope®was handy and useful to determine the border between DCSO lesion andhealthybone.The free fibula flap under the minocycline-derived bone fluorescence by using a VELscope®offered a good quality of mandibular bone and the successful management of an advanced and refractory DCSO.

** Key words:**Fluorescence-guided bone resection, fibular free flap, osteomyelitis of the mandible, diffuse chronicosteomyelitis, VELscope®.

## Introduction

Diffuse chronic sclerosing osteomyelitis (DCSO) of the jaw, first described by Marzola (1,2), results from an immunologic reaction to microbial stimuli, such as bacterial toxins and infection, but this hypothesis was not supported by bacteriologic and histologic findings. The management of DCSO thus remains challenging (3). Although a variety of conventional treatments have been tried for DCSO, and a small proportion of patients have had long-term relief of symptoms, most cases ofDCSO are refractory to treatment. Once the disease process becomes chronic, the general consensus is that the most effective treatment for DCSO of the jaw is a combination of antimicrobial therapy and surgical debridement. However, the treatment often requires several protracted courses of antibiotics and multiple surgical interventions, because the etiology and pathogenesis remain poorly understood (4,5). However, currently, the surgical therapy is only loosely standardized for the patients who are refractory to several conservative treatments. On the other hand, these surgical treatments are controversial, because even after the aggressive surgical treatment it may become worse. One of the reasons is the difficulty to define the margins of the unhealthy bone more precisely, because no suitable imaging modalities exist to determine the resection area and the excision of the lesion is insufficient and then would be recurrence. To date, intraoperatively, it is not possible to visualize this feature more easily, and no successful surgical treatment protocols have been published. Therefore, we considered that determination of the border between healthy bone and unhealthy bone was important. Minocycline, a tetracycline antibiotic, is incorporated in healthy bone and has the effect of inducing visual fluorescence retention. Due to this incorporation, it can be detected by its fluorescence (6). To date, tetracycline bone fluorescence has been introduced as the surgical therapy of several bone diseases in some hospital facilities (7). We then reported our successful experience with a patient who underwent surgical treatment with radical resection of the hemimandible including the condyle with immediate reconstruction with a free fibula flap under minocycline bone fluorescence imaging by Visually Enhanced Lesion Scope (VELscope®). The histopathological investigation ofthe fluorescence area of the resected specimen was also shown.

## Case Report

**Figure 1:**
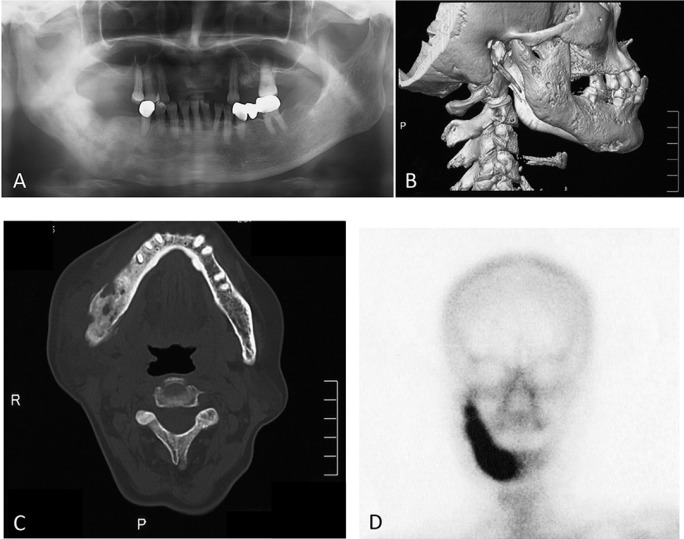
Panoramic radiography A) and Computed tomography B,C) show bone sclerosis with scattered osteolysis of the ascending ramus. Technetium 99m bonescintigram D) shows enhanced uptake in only the right part of the mandible.

Fluorescence guided bone resection was performed after written informed consent in the female patient. A 66-year-old female was referred to the Department of Oral and Maxillofacial Surgery, Kyushu Dental University Hospital, with a pain and swelling around the mandibularangleon the right side, which firstly occurred two years before and presented with history of recurrent pain and swelling over the right side of mandible. The patient reported endodontic treatment on the right lower second molar and chemotherapy of antibiotics were performed about 1 month ago by a general dentist, but it failed to improve the symptoms. The panoramic radiography showed change in bone density in the region of the ill-defined osteosclerotic change of the right mandible (Fig. 1A). Computed tomography of the mandible showed localized widening and sclerosis of the right hemimandible (Fig. 1B,C) and bone scintigram (technetium 99m) showed enhanced uptake in only the right side of the mandible (Fig. 1D).

**Figure 2:**
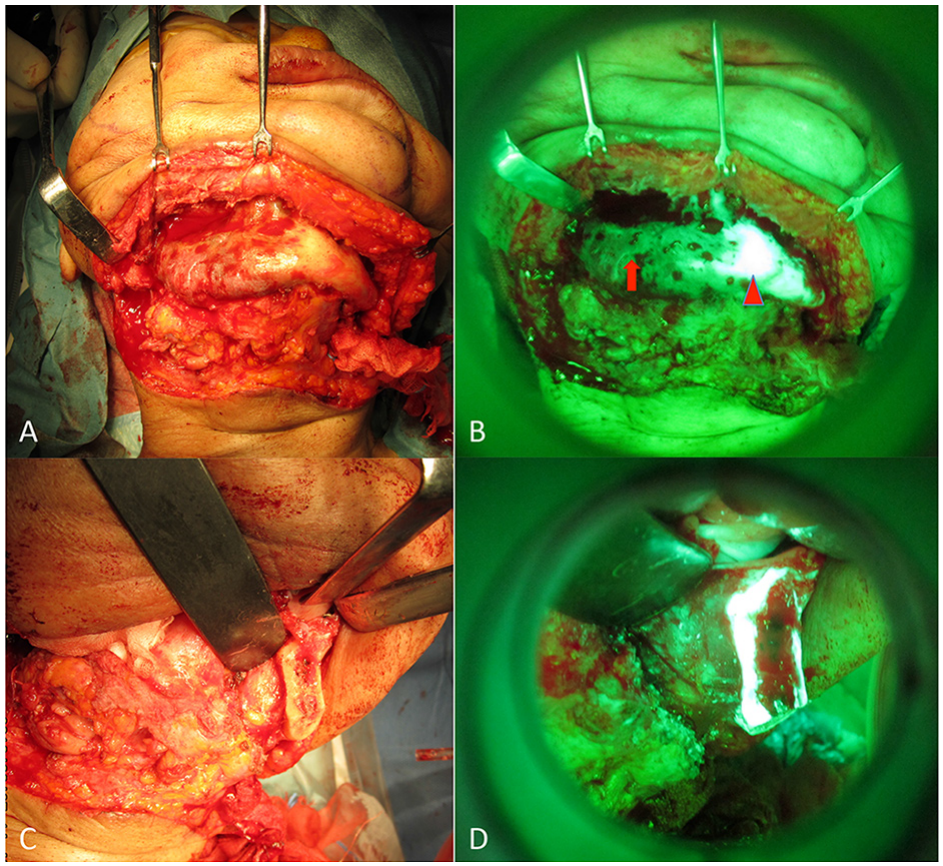
Intraoperative clinical picture A) after subperiosteal exploration. The extent of the unhealthy bone became obvious by use of VELscope® B) (arrowhead: Detected area show healthy bone. arrow: Undetected area show unhealthy bone) Intraoperative clinical picture of resected section C) and fluorescence picture D) after fluorescence-guided bone resection.

Preoperatively, this patient received minocycline 100 mg twice a day for 1 month. Using a VELscope® (LED Dental Inc., Burnaby, BC, Canada), a certified medical device (no. 41315446) approved by the American Dental Association for the detection of mucosal tissue abnormalities (CDT D0431), minocycline-derived bone fluorescence was visualized intraoperatively under blue excitation. In this surgical approach, hemimandibulectomy was conducted under fluorescence guidance (Fig. 2. A-D). Surgical treatment consisted of a complete resection of the right ascending ramus of the mandible, including the condyle, and immediate reconstruction with a microsurgically anastomosed fibula flap from the left side. Postoperative healing was uneventful without major functional or aesthetic impairment. The patient showed normal mouth opening and a standard occlusion. There was no relevant donor site morbidity. A postoperative panoramic radiograph showed the reconstructed ascending ramus to be in the correct position. The clinical and radiographic follow-up showed no signs of a recurrence.

**Figure 3:**
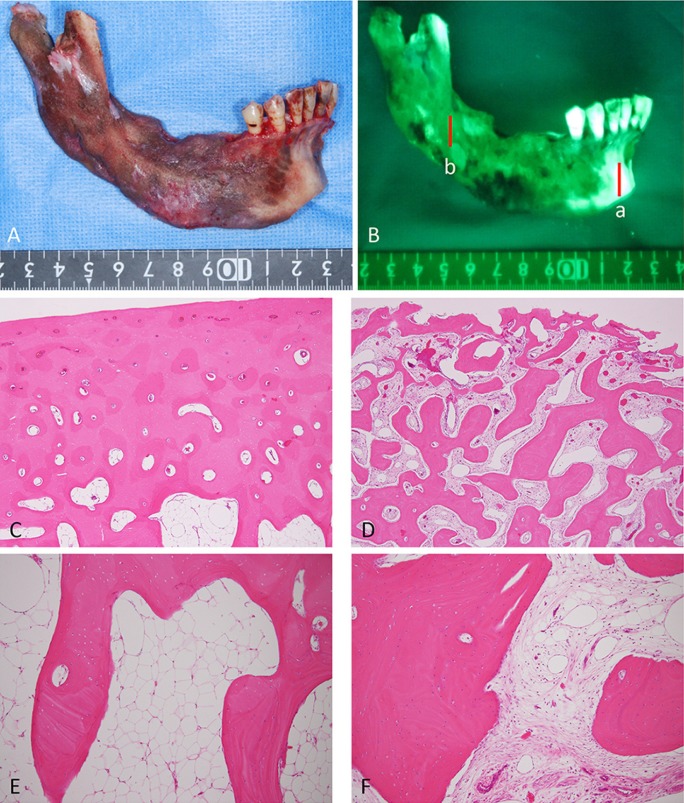
Clinical picture and fluorescence picture of the postoperative resected mandible A). VELscope® findings of the resected mandible B). Histopathological findings of the following section; a and b C-F). (sectiona; C and E, section b; D and F, C,D; ×40 magnification, E,F; ×100 magnification).

We showed the macroscopic findings and the VELscope® findings of the resected mandible and compared with each findings (Fig. 3A,B). The histopathological findings of the region of fluorescent positive showed healthy bone covered with thickcortical bone tissue and consisted of age-appropriate fatty marrow without inflammation, fibrosis, norhyperemia. Additionally, there were lining of a few intact osteoblasts and osteocytes in healthy bone (Fig. 3C,E). On the other hand, postoperative histopathological findings of the chronic sclerosing osteomyelitis lesion; non-fluorescence area showed little compact bone tissue in the substantial portion of cortical bone per low-power field (Fig. 3D) and dense, irregular bony trabeculae bordered by an active layer of osteoblasts and intermingled fibrous tissue with mild lymphocytic infiltration per high-power field (Fig. 3F). The non-fluorescent moietyand the DCSO lesions had histopathological correlations. Additionally, microbiologic culture from the biopsy specimen was negative for microorganisms.

## Discussion

Despite the development of various new antibiotics and anti-inflammatory drugs, DCSO of the mandible often does not respond to medical treatment (8-10). Because recurrent symptoms may persist for years, still some patients require surgical resection of the mandible. Although demarcation of unhealthy bone is of crucial importance in the therapy of the DCSO of the jaw, at present, no effective and no useful modalities allow the precise intraoperative identification of affected bone. Indeed, if the unhealthy bone area can be undetected, residual unhealthy bone results in a progression of the osteomyelitis or recurrence of DCSO.

VELscope®is a reusable light source that emits a cone of light in the blue spectrum (400-460 nm) into the oral cavity, causing fluorophores in the oral tissue to excite and fluoresce. In particular, VELscope® has been widely used in the diagnosis of precancerous lesions, such as leukoplakia, carcinoma in situ or squamous cell carcinoma of the oral cavity using autofluorescence imaging (11). On the other hand, tetracycline bone labeling is a diagnostic tool routinely applied to address diverse questions of bone metabolism at the microscopic level (12). However, to our knowledge, there are few reports about the intraoperative use of macroscopic tetracycline fluorescence imaging of healthy versus unhealthy bone in patients with DCSO. This is the report to investigate about DCSO histopathologically and discuss the efficacy of the VELscope® (13).

In this case, the typical fluorescence pattern in osteomyelitis showed a really weak signal in the cortical bone. Due to this new bone formation, tetracycline incorporation is evident in the respective regions resulting in the typical fluorescence pattern. The histopathological findings confirmed that the non-fluorescent moiety was closely correlated with the DCSO lesions.VELscope®is a suitable tool to visualize areas of the unhealthy bone by visual fluorescence loss in patients with DCSO. As demonstrated, visual fluorescence loss in the unhealthy areas of the bone is useful as a guide for fluorescence-guided bone resection with relevant cli-nical interpretation. Generally, surgical procedures are indicated in almost all patients with DCSO who show resistance to the conventional treatment with antibiotics. Using the VELscope®device, excellent results can be reached regarding complete resection of unhealthy bone areas, and postoperative outcome after wound closure and wound healing.

The most commonly affected regions with osteomyelitis of the mandible include the angle and body of the mandible (14). The occurrence has been reduced since the advent of antibiotics and improved dental hygiene. Currently, patients at risk of developing osteomyelitis of the mandible including those who have undergone radiotherapy to the head and neck region, are immunocompromised, are heavy smokers and drinkers, are on immunosuppressive therapy, or suffer from uncontrolled diabetes (9,14). The most common presenting symptoms include facial swelling, pain, trismus and hypoesthesia of mentum. Once the disease process has been present for a month and is refractory to conventional treatments, such as oral antibiotics, it is termed chronic. Once the disease process becomes chronic, the general consensus is that the most effective treatment for chronic osteomyelitis of the mandible is a combination of antimicrobial therapy and surgical debridement (15). However, the management often requires several protracted courses of antibiotics, at least 1 hospital admission, and multiple surgical interventions.

We reported our successful experience with a patient who underwent surgical treatment with radical resection of the hemimandible including the condyle with immediate reconstruction with a free fibula flap under minocycline bone fluorescence imaging by VELscope®. We recommend the minocycline-derived bone fluorescence by using a VELscope® thatis a valuable and useful techniqueto distinguish healthy bone from unhealthy bone in the treatment of refractory DCSO. Microvascular reconstruction with a fibular free flap under the minocycline-derived bone fluorescence technique by using a VELscope® should be considered as a treatment option in the management of DCSO of the mandible because of the consistency with the histopathological findings.
